# Why Are Stroke Rehabilitation Trial Recruitment Rates in Single Digits?

**DOI:** 10.3389/fneur.2021.674237

**Published:** 2021-06-08

**Authors:** Shashwati Geed, Preethy Feit, Dorothy F. Edwards, Alexander W. Dromerick

**Affiliations:** ^1^Center for Brain Plasticity and Recovery, Department of Rehabilitation Medicine, Georgetown University Medical Center, Washington, DC, United States; ^2^MedStar National Rehabilitation Hospital, Washington, DC, United States; ^3^Department of Kinesiology and Occupational Therapy, University of Wisconsin, Madison, WI, United States; ^4^Department of Neurology, Georgetown University Medical Center, Washington, DC, United States

**Keywords:** prospective study, rehabilitation, clinical trial, stroke, trial design

## Abstract

**Background:** Recruitment of patients in early subacute rehabilitation trials (<30 days post-stroke) presents unique challenges compared to conventional stroke trials recruiting individuals >6 months post-stroke. Preclinical studies suggest treatments be initiated sooner after stroke, thus requiring stroke rehabilitation trials be conducted within days post-stroke. How do specific inclusion and exclusion criteria affect trial recruitment rates for early stroke rehabilitation trials?

**Objectives:** Provide estimates of trial recruitment based on screening and enrollment data from a phase II early stroke rehabilitation trial.

**Methods:** CPASS, a phase II intervention trial screened ischemic stroke patients in acute care (18-months, *N* = 395) and inpatient rehabilitation (22-months, *N* = 673). Patients were stratified by upper extremity (UE) impairment into *mild* (NIHSS motor arm = 0, 1); *moderate* (NIHSS = 2, 3); *severe* (NIHSS = 4) and numbers of patients disqualified due to CPASS exclusion criteria determined. We also examined if a motor-specific evaluation (Action Research Arm Test, ARAT) increases the pool of eligible patients disqualified by the NIHSS motor arm item.

**Results:** CPASS recruitment in acute care (5.3%) and inpatient rehabilitation (5%) was comparable to prior trials. In acute care, a short stay (7–17-days), prior stroke (13.5% in *moderately*; 13.2% in *severely* impaired) disqualified the majority. In inpatient rehabilitation, the majority (40.8%) were excluded for “too mild” impairment. The next majority were disqualified for reaching inpatient rehabilitation “too late” to participate in an early stroke trial (15% in *moderately*; 24% in *severely* impaired). Mean ARAT in the “too mild” showed significant impairment and potential to benefit from participation in select UE rehabilitation trials.

**Conclusions:** Screening of ischemic stroke patients while they are still in acute care is crucial to successful recruitment for early stroke rehabilitation trials. A significant proportion of eligible patients are lost to “short length of stay” in acute care, and arrive to inpatient rehabilitation “too late” for an early rehabilitation trial. Additional screening of mildly impaired patients using a motor function specific scale will benefit the trial recruitment and generalizability.

**Trial Registration Number:** http://www.clinicaltrials.gov Identifier: NCT02235974.

## Introduction

Stroke rehabilitation trials have conventionally focused on individuals whose motor recovery has plateaued at more than 3–6 months post-stroke (1–5) so that spontaneous post-stroke recovery does not confound intervention-related recovery (6). However, preclinical studies increasingly suggest that post-stroke rehabilitation intervention trials need to be conducted within days of stroke onset for better neuromotor outcomes (2, 7–9). In traditional stroke rehabilitation trials, investigators have a long window of recruitment often lasting up to several years post stroke because patients who are more than 6-months post stroke are included. Additionally, rehabilitation trialists have conventionally recruited from outpatient stroke clinics and community centers that offer multiple opportunities with repeated contact to enroll the same individuals.

To enable acute (<7 days) and early-subacute (8–90 days post stroke) trials (10, 11), rehabilitation trial recruitment methods need to adapt by shifting into the acute care and inpatient rehabilitation settings where patient stays are limited for 2 weeks post stroke on average (12). Thus, early stroke rehabilitation trialists have a brief window of identifying and enrolling eligible patients in trials. This brief period coincides with a particularly confusing time in patients' lives given their recent stroke diagnosis and the long commitment required of them to participate in a stroke rehabilitation trial with a typical follow up at 12-months post randomization (5, 7, 13–15). Even small efficiencies in patient screening and enrollment in this scenario can make large differences in the eventual trial recruitment rates and costs. However, there are no data at present that give reliable estimates of how trial inclusion and exclusion criteria affect the pool of eligible patients in a US healthcare setting for an early stroke rehabilitation trial.

Lasagna's law (16) notes that “the incidence of any disease decreases sharply as soon as a clinical trial begins and returns to its original level as soon as the trial is completed.” No matter how conservative one is about their recruitment goals, it is difficult to recruit study participants according to expectations. Incorrect estimates of trial recruitment lead to delays in study completion, abandoned studies, and mismanagement of research funds (17). Underpowered studies increase the probability of type II errors affecting study integrity and validity. Inadequate trial inclusion/exclusion criteria negatively affect generalizability and internal validity of the study. Thus, reliable estimates of trial recruitment rates are central to any trial's planning and logistics.

Here, we report screening and enrollment data from the phase II Critical Periods After Stroke Study (CPASS), an early stroke rehabilitation trial designed to identify optimal timing of upper extremity (UE) motor rehabilitation after stroke (7, 18). CPASS recruited individuals within 30 days of stroke and followed participants up to 12 months post randomization. CPASS screening data are from an urban safety-net acute-care hospital and an inpatient rehabilitation setting that used StrokeNet (19) resources. We also report the trial inclusion/exclusion criteria that most affected trial recruitment rates to help future investigators re-evaluate their own trial inclusion and exclusion to aid trial recruitment. Finally, UE stroke rehabilitation trial screening often relies on a *prescreen* (an easily available measure of motor function from medical history or charts to filter the patients for evaluation with the full trial inclusion/exclusion criteria), e.g., scores on the National Institutes of Health (NIHSS) Stroke Scale items (2, 20). Using CPASS data, we asked if stroke rehabilitation trial recruitment rates would benefit from using a motor function-specific screening tool over the commonly used NIHSS. This is a first report to our knowledge that highlights the typical characteristics of patients recruited or excluded for an early-subacute stroke neurorehabilitation trial in the US healthcare setting.

## Methods

Study procedures were approved by the Institutional Review Board at the MedStar Health Research Institute. The trial was registered at http://www.clinicaltrials.gov (Identifier: NCT02235974). Registry data from inpatients at MedStar Washington Hospital Center and MedStar National Rehabilitation Hospital (NRH) were used. The registry identifies potential participants for post-acute stroke studies at 24–48 h after admission. Data from neuroimaging-confirmed ischemic stroke survivors were extracted; additionally, data on the earliest available National Institutes of Health Stroke Scale (NIHSS) score, demographics, thrombectomy status, tissue plasminogen activator (tPA) administration, duration of stay, and discharge location were extracted. Patients entering inpatient rehabilitation at NRH are evaluated using the Action Research Arm Test (ARAT), a UE motor function specific assessment (21–24) as part of their intake evaluation; these ARAT scores were also extracted. We report CPASS screening data for an 18-month (02/2016 – 07/2017) duration from acute care, and 22-month duration (06/2015 — 04/2017) from the inpatient rehabilitation setting.

UE rehabilitation trials typically stratify enrolled participants using severity of UE motor impairment (2, 5, 7, 14, 25). Therefore, to examine the effect of motor severity on trial eligibility rates, we used NIHSS arm motor score to categorize UE impairment into *mild* (NIHSS = 0, 1), *moderate* (NIHSS = 2, 3), or *severe* (NIHSS = 4) impairment (12).

To examine the impact of other trial inclusion/exclusion criteria on trial qualification rate, we used the medical, disability, and social support criteria from CPASS (Table 1). CPASS criteria are similar to multiple major stroke rehabilitation trial inclusion/exclusion criteria, including, VECTORS (2), ICARE (5), EXCITE (14), and LEAPS (4), and TRANSPORT-2.

**Table 1:** CPASS trial inclusion and exclusion criteria.

**Inclusion criteria**
1. Ischemic or hemorrhagic stroke (with confirmatory neuroimaging) within 28 days of admission to inpatient rehabilitation 2. Age ≥21 years 3. Able to participate in first study-related treatment session within 30 days of stroke onset 4. Able to participate in all study-related activities, including 1 year follow up and blood draws 5. Persistent hemiparesis leading to impaired UE function as indicated by a score ≥1 on the NIHSS motor arm score, and motor impairment judged clinically appropriate as defined by one or more of the following: a. Proximal UE voluntary activity indicated by a score of ≥3 on the upper arm item of the motor assessment scale; wrist and finger movements are not required b. Manual muscle test (MMT) score ≥2 on shoulder flexion and either elbow flexion or extension or c. Active range of motion (AROM) to at least 50% of range in gravity eliminated position for shoulder flexion or abduction, and for any of the following motions: elbow flexion, elbow extension, wrist flexion, wrist extension, finger flexion or finger extension 6. Score of ≤8 on the Short Blessed Memory Orientation and Concentration scale 7. Follows 2-step commands 8. No upper extremity injury or conditions that limited use prior to the stroke 9. Pre-stroke independence: Modified Rankin Scale score of 0 or 1
**Exclusion criteria**
1. Inability to give informed consent. 2. Prior stroke with persistent motor impairment or other disabling neurologic conditions such as multiple sclerosis, Parkinsonism, ALS, dementia requiring medication. 3. Rapidly evolving motor function. 4. Clinically significant fluctuations in mental status in the 72 hours prior to randomization. 5. Hemispatial neglect as determined by an asymmetry >3 errors on the Mesulam symbol cancellation test. 6. Not independent prior to stroke (determined by scores of <95 on Barthel Index or >1 on modified Rankin scale. 7. Dense sensory loss indicated by a score of 2 on NIHSS sensory item. 8. Ataxia out of proportion to weakness in the affected arm as defined by a score ≥1 on the NIHSS limb ataxia item. 9. Active or prior (within 2 years) psychosis. 10. Active or prior (within 2 years) substance abuse. 11. Not expected to survive 1 year due to other illnesses (cardiac disease, malignancy, etc.). 21. Received UE botulinum toxin within 6 months (other meds do not exclude).

Even a single exclusion criterion will put the patient out of the “qualified” pool of participants during screening. We followed a similar strategy to quantify patients excluded from the pool of eligible patients, but it is likely a patient in a given category has multiple exclusionary characteristics. Further, not all patients qualifying for the trial will eventually be enrolled. We therefore report the actual enrollment numbers in CPASS from acute care and inpatient rehabilitation to give trialists an estimate of the total numbers of patients screened for the number enrolled in the trial. Specifically, the results report the numbers of patients excluded due to each trial exclusion criteria from the acute care and inpatient rehabilitation screening. To determine the added benefit of using a UE motor-function specific scale on trial qualification rate, we examined the ARAT, in patients that were disqualified from CPASS due to their UE being “too mild” (NIHSS motor arm score <1). Descriptive statistics on the ARAT scores in patients with NIHSS motor arm score <1 were computed.

## Results

### In Acute Care, Short Length of Stay and Prior Stroke Lead to the Most Exclusions

We identified 395 ischemic stroke survivors with an available NIHSS motor arm score from the acute care registry over the 18-month screening duration. Mean NIHSS total score was 9.1 ± 9.3. Of these, 46.6% were male, and 77.2% identified as African American (12.3% as White, and 1.4% as Asian with the rest Unknown or Refused to identify). Of the 395 patients, 21.2% received tPA, and 8.2% received a thrombectomy. Examining the degree of motor impairment, 64.8% had mildly impaired UE (NIHSS motor arm = 0 or 1), 18.3% had moderately impaired UE (NIHSS motor arm = 2 or 3), and 15.6% had severe UE impairment (NIHSS motor arm = 4). Overall, 5.3% of the 395 individuals identified in screening eventually enrolled in CPASS from acute care.

To determine the average window of time to enroll patients, we examined patients' average length of stay. The length of stay in acute care and discharge locations for the three groups (mild, moderate, and severely impaired) is shown in Table 2. Length of stay correlated with degree of UE impairment, mild impairment = 6.9 ± 6.9 days, moderate = 13.6 ± 15.4 days, and severe = 17.2 ± 13.1 days. Only 34% of the mildly impaired, 49% of the moderately impaired, and 31% of the severely impaired patients were discharged to an inpatient rehabilitation facility, which opens a second window to recruit these patients if the trialist implements screening resources at acute care and inpatient rehabilitation. 83/395 ischemic stroke patients screened in this acute care report were admitted to inpatient rehabilitation at NRH and are included in the data on inpatient rehabilitation screening data as well to maintain integrity of the independent screening numbers at acute care and inpatient rehabilitation. A majority (54%) were discharged home or to a skilled nursing facility. Unless a trial budgets for outreach to enroll patients discharged home, this large majority of potentially eligible patients may be entirely lost to trial enrollment if not recruited during their short hospital stay (6–17 days).

**Table 2:** Length of Stay and Discharge locations in Acute Care and Inpatient Rehabilitation.

	**Mild**		**Moderate**		**Severe**	
	**Acute**	**Inpatient**	**Acute**	**Inpatient**	**Acute**	**Inpatient**
Length of stay (days)	6.9	13.7	13.6	20.5	17.2	32.1
Discharge home, no service (%)	36.7	45.9	10.7	32.5	6.2	16.3
Discharge home, home health (%)	11.3	5.8	4.0	2.5	6.2	4.9
Discharge home, outpatient OT/PT (%)	5.1	30.2	1.3	37.5	0.0	28.5
Acute inpatient rehabilitation (%)	34.0	0.7	49.3	0.8	30.8	2.4
Subacute rehabilitation/SNF (%)	9.4	13.3	17.3	19.2	32.3	35.0
Another acute care hospital (%)	0.0	3.6	2.7	6.7	4.6	12.2
Hospice (%)	0.8	0.2	4.0	0.8	0.0	0.0
Death (%)	1.6	0.2	9.3	0.0	20.0	0.8
Other (%)	0.0	0.0	1.3	0.0	0.0	0.0

**Figure 1:**
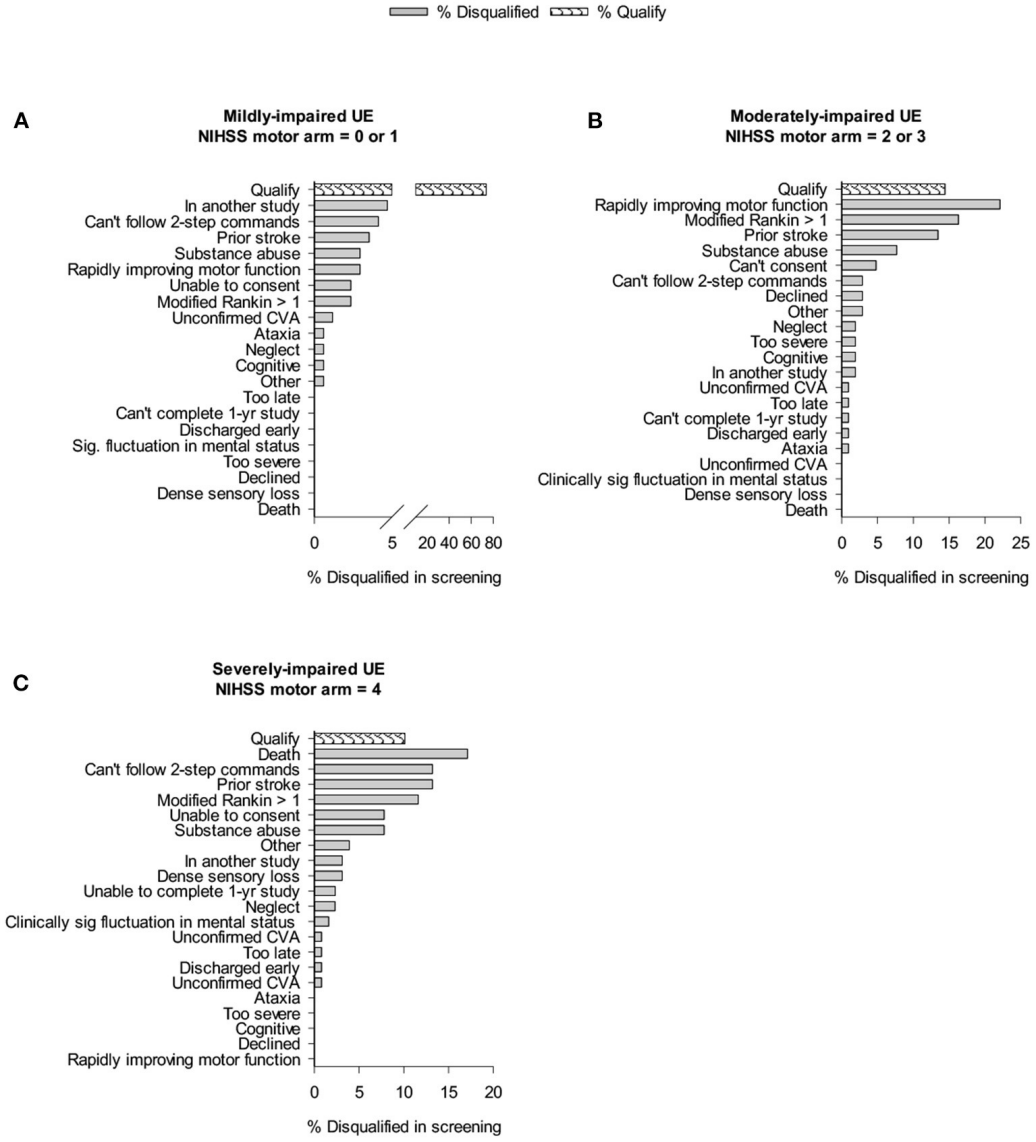
Cumulative impact of study criteria on trial recruitment rates in acute care. Bar graphs show % of individuals screened that were disqualified because of given trial exclusion criteria. “Qualify” shows % of those screened who qualified for CPASS-like trial. Cumulative effects of the exclusion criteria result in a progressively smaller pool of patients that will qualify for a trial.

Figure 1 shows the impact of trial exclusion criteria on the pool of eligible patients from the mild, moderate, and severe UE impairment groups. In patients with mild impairment (Figure 1A), 73.5% would potentially qualify for motor studies recruiting subjects with NIHSS motor arm score = 0 or 1. Examination of general medical, disability, and social criteria showed that main reasons for ineligibility include “in another study” (4.7%), and “inability to follow 2-step commands” (4.1%). In patients with a moderately-impaired arm (Figure 1B), a mere 14.4% (3.7% of *all* ischemic stroke patients) would qualify for a CPASS-like UE trial. In this moderately-impaired cohort, “rapidly improving motor function” (22%), “disabled prior to stroke” (16.3%), and “prior stroke with persistent impairments” (13.5%) were the most frequent exclusions. In the severely impaired group (Figure 1C), 89.9% of patients were eliminated by medical, disability, and social criteria. Patients with severe UE impairment were most frequently excluded by prior stroke (13.2%), or pre-stroke disability (11.6%). Overall, 64% of patients with moderately-impaired (and 70% of those with severely impaired) UE's were excluded not by stroke, but rather inability to consent, substance abuse, prior stroke, pre-stroke disability, or rapid clinical improvement.

### At Inpatient Rehabilitation, Arriving Too Late, Mild Impairment, and Prior Stroke Lead to the Most Exclusions

We identified 673 ischemic stroke survivors with an available NIHSS motor arm score during the 22-month screening for CPASS. Mean NIHSS total score was 6.6 ± 5. Of these 53.9% were male, 65.1% identified as African American, 16.8% as White, 0.9 and remaining as Asian, Other, or Refused to identify. Overall, 5% of the 673 individuals screened enrolled in CPASS.

Examining the degree of motor impairment, 62.1% showed Mild, 19.7% Moderate, and 18.2% showed severe UE impairment. Average length of stay correlated with the degree of motor impairment; patients with mild impairment were inpatients for 13.7 ± 13.4 days, moderate impairment for 20.5 ± 15.6 days, and patients with severe impairment were inpatients for 32.1 ± 20.4 days. Discharge locations by degree of motor impairment are shown in Table 2.

**Figure 2:**
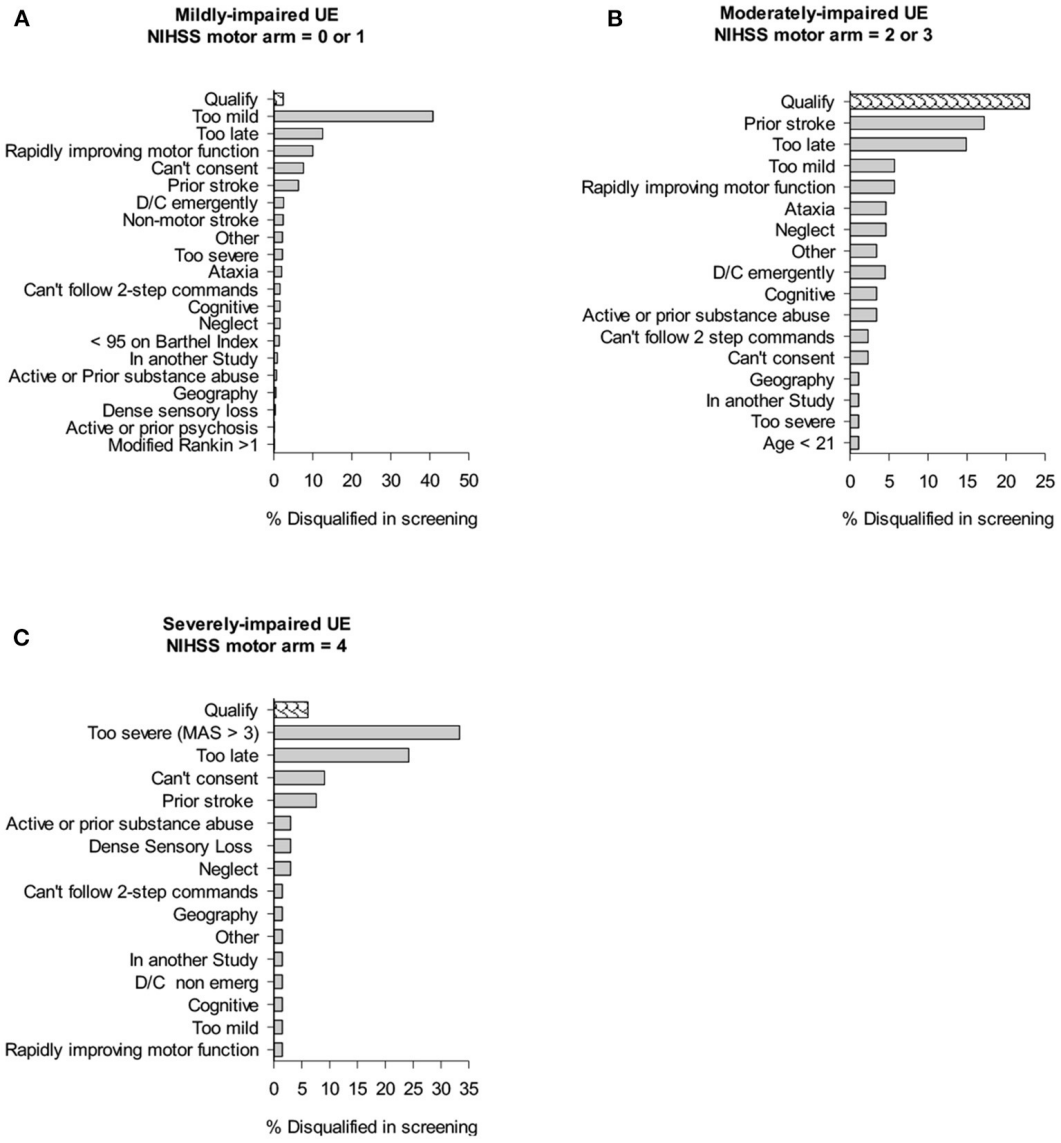
Cumulative impact of study criteria on trial recruitment rates in inpatient rehabilitation. Bar graphs show % of individuals screened that were disqualified because of given trial exclusion criteria. “Qualify” shows % of those screened who qualified for CPASS trial.

Figure 2 shows the impact of trial exclusion criteria on the available pool of qualified patients for the UE trial. Of all the mildly impaired participants, 40.8% of patients in inpatient rehabilitation were excluded because their impairment was too mild for the subacute intervention study (NIHSS motor arm score = 0 or 1). The next largest group (12.5%) was disqualified for being “too late,” i.e., they arrived at inpatient rehabilitation too late and would not receive the first study-related treatment session within 30-days of stroke onset. Ten percentage of mild inpatients were disqualified because their arm was recovering too rapidly for the context of this trial, e.g., the chart sheet recorded NIHSS motor arm item score = 2 but in-person screening showed little to no deficit in the UE. The next largest group, 7.6% were disqualified for being unable to consent because of language issues, inability to understand the consent process, or aphasia. Lastly, “prior stroke” disqualified 6.3% of the patients. Of all the moderately impaired patients (NIHSS = 2 or 3), the largest percentage (17.2%) were disqualified because of a prior stroke. The next largest group (14.9%) were disqualified because they arrived at inpatient rehabilitation “too late.” Of all the severely impaired patients, the largest disqualification rate (33.3%) came from patients being too severe for the CPASS trial (enrolling mild-moderately impaired), and 24.2% of patients were disqualified for being “too late” to inpatient rehabilitation.

### Screening With ARAT Instead of NIHSS Motor Arm Item Benefits Trial Recruitment

**Figure 3:**
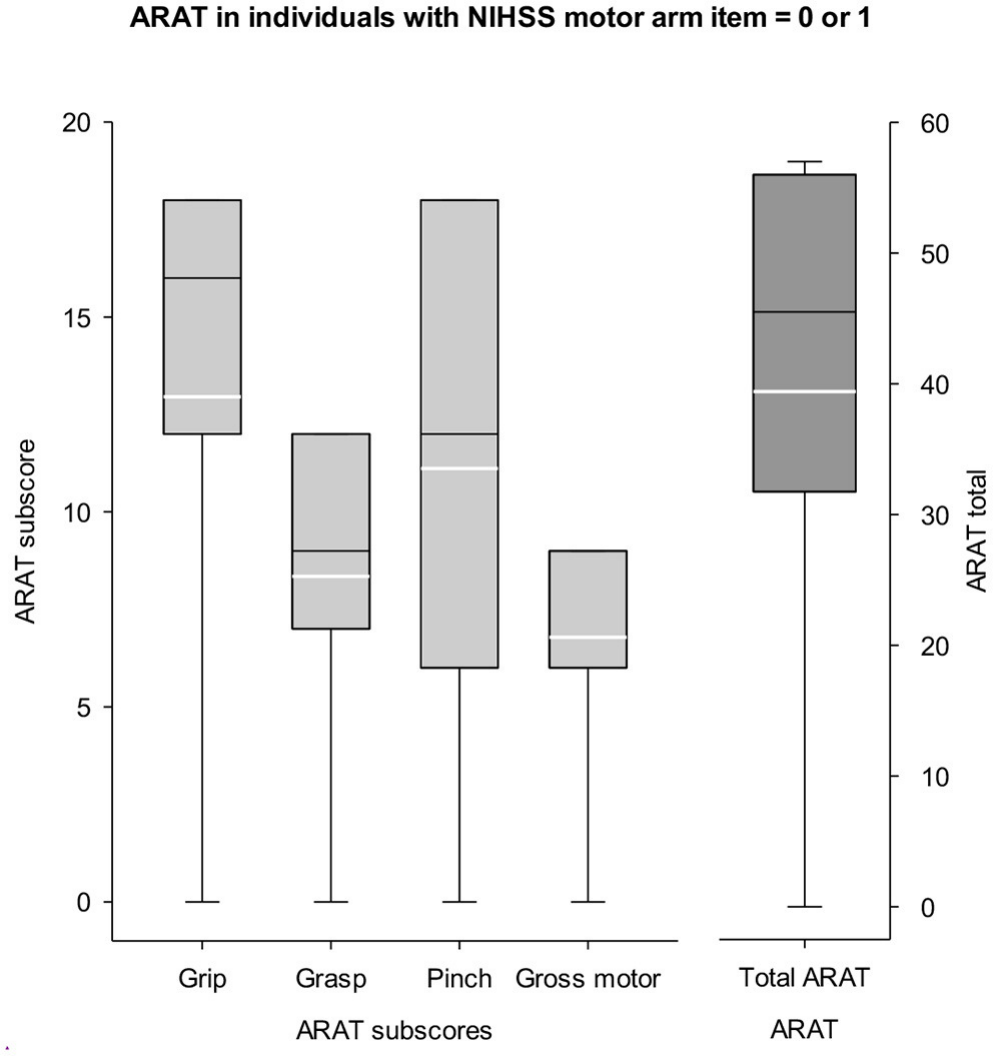
ARAT scores in mildly impaired patients (NIHSS = 0 or 1). Left y-axis represents the ARAT sub scores for *grasp, grip, pinch, and gross*. Right y-axis shows the scale for ARAT total score (max = 57). Participants typically disqualified from a study seeking moderately impaired UE (NIHSS motor arm < 1) show potential for clinically meaningful recovery given their mean ARAT score = 39/57. White lines in boxplots show the mean score. There is some benefit in completing a motor specific screening like the ARAT for UE stroke rehabilitation trials to engage a subset of patients typically disqualified with the NIHSS screening criteria.

A large percentage of all patients admitted to acute care (64.8%) and inpatient rehabilitation (62.1%) that were screened for CPASS were disqualified for what would be classified as a “mildly impaired arm (NIHSS = 0 or 1).” These patients would be automatically disqualified from UE stroke rehabilitation trials if screening were completed using only the NIHSS motor arm item and CPASS-like rehabilitation trial Inclusion/Exclusion criteria. Given the large percentages disqualified due to a “too mild” score on NIHSS motor arm item, we evaluated if there is any added benefit in numbers of patients qualifying for a UE stroke study if screening were done using a dedicated motor function specific scale like the ARAT. The distribution of ARAT scores in a random selection of 389/438 patients screened at inpatient rehabilitation is shown in Figure 3. Mean ARAT score for the affected UE was 39.41±18.6, and 52.7±9.9 for the unaffected UE (max score = 57).

## Discussion

Of the 395 ischemic stroke patients screened in acute care over 18 months, 5.3% were enrolled in CPASS. On the inpatient rehabilitation side, of the 673 ischemic stroke patients screened over 22 months, 5% were enrolled in CPASS. These single-digit recruitment rates are comparable with major stroke rehabilitation trials. ICARE (5) recruiting patients in the subacute stage screened 11,051 patients to randomize 361 patients (recruitment rate of 3.2%), EXCITE recruiting patients in the subacute phase (14, 26) screened 3,626 patients to randomize 222 patients (6.1%), AVERT (17) screened 25,237 patients in the hyperacute stage, to randomize 2,104 (8.3%). Thus, trial recruitment rates have remained in the single-digits for a majority of large multisite stroke rehabilitation trials.

Randomized controlled trials are the gold standard for evaluating treatment effectiveness. Major stroke rehabilitation trials so far have shown only marginal effectiveness in improvement of motor function compared to the standard of care (2, 5, 27–36). In the US, a stroke occurs every 40 sec (37); most individuals survive but with long-term motor impairments that seriously limit independent living. Nearly two thirds of stroke survivors are unable to use their affected arm even at 6 months post stroke (38–40). Thus, there is an urgent need for effective UE neurorehabilitation. Single-digit recruitment rates significantly slow down translation of preclinical to phase II/III studies while increasing the costs of stroke rehabilitation trials. Stroke trial recruitment rates have remained unchanged, in single digits between 1990 and 2014 (41) necessitating a systematic examination of how specific trial inclusion/exclusion criteria affect recruitment.

Recruitment logistics for acute-and early subacute stroke rehabilitation trials (<30-days post) contrast sharply with conventional stroke rehabilitation trials typically conducted in the chronic phase (>6-months post-stroke). This early trial recruitment is challenging because patients are admitted for short duration (6–17 days). Patients' medical status changes rapidly during this time including their degree of motor arm impairment as shown by the large fractions of patients excluded because of a rapidly recovering UE. Correctly estimating if future motor impairment will be suitable for the trial, given patients' current motor function status is difficult (42, 43). This is especially relevant in the neurorehabilitation where interventions involve daily sessions for weeks and follow-ups at 12-months post-stroke (5, 7, 13–15, 26, 44).

Given the unique challenges in recruiting for neurorehab studies early after stroke, we presented prospectively collected estimates of how specific trial inclusion and exclusion criteria affect trial recruitment rates. Based on our data, we also provide recommendations for best practices in optimizing trial inclusion/exclusion criteria to strike a balance between trial generalizability and maintaining a homogenous enough sample in the trial (Box 1). Importantly, multisite trials need to conduct thorough screening of potential recruitment sites prior to commencing trials such as CPASS to ensure that the site has adequate patient throughput and to identify potential site-specific barriers to recruitment (2). Patients' length of stay, transfer from acute care to inpatient rehabilitation or community rehabilitation services affects access to patients for recruitment.

Box 1 **Recommendations for best practices in screening for acute and early subacute trials.**

1. Prescreening with a motor function specific tool like SAFE or ARAT may be better than NIHSS motor arm item alone, especially for the mildly impaired patients as it leads to a large percentage of exclusions.
2. Patients excluded for being too mild based on NIHSS criteria alone should be considered for inclusion in trials as their mean ARAT score (39.4±18.6) is substantially lower than the ARAT ceiling of 57. The difference between mean ARAT in mildly-impaired and ARAT's ceiling is greater than the ARAT MCID, suggesting these patients are likely to benefit from participating in the trial, and show clinically meaningful improvement.
3. Screening and recruitment efforts must begin in acute care as a large percentage arrive to inpatient rehabilitation too late to be enrolled in early stroke trials.
4. Prior strokes lead to a large percentage of exclusions. Trialists need a pragmatic definition for which patients should be excluded given the large percentages of second strokes. For phase II/III motor function intervention trials, “prior stroke without residual motor impairment” may be acceptable; translational neurophysiological studies may need prior strokes to be fully excluded.
5. Multisite trials selecting sites for trials need to evaluate screening data from local studies to determine site-specific barriers to recruitment, transfer from acute care to inpatient rehabilitation or community services which may enhance recruitment potential of a site.

### In Acute Care: Short Length of Stay, mRS>1, and Prior Stroke Limit Recruitment

CPASS screening data from the acute care setting showed patients' length of stay varied from 6 days for the mildly impaired to 17 days for the severely impaired patients. On average, the moderately impaired were admitted for 13.6 days. A majority of these moderately-impaired (49%) were discharged to Inpatient rehabilitation, which opened another window to recruit them for stroke trials, but the remaining 32% discharged to home (15%) or to a skilled nursing facility (17%) would be lost to recruitment forever unless a trial developed extensive outreach resources to recruit them from the community. These lengths of stay and discharge data are specific to an urban safety net hospital in the US where CPASS screening was conducted. Similar exhaustive data on recruitment for rehabilitation trials from across the US are missing at this time. Recently [29)], screening for acute stroke patients at the University Hospital Zurich in Switzerland reported on the eligibility criteria for a comparable UE trial. The typical length of stay (mean = 8; range 4–12 days; not stratified by severity as in the present report) was consistent with the brief length of stay we found with CPASS screening. It is critical for early stroke rehabilitation trials to develop efficient screening methods to rapidly identify eligible patients at the earliest time point after stroke. Other exclusion criteria that led to most exclusions in acute care included “rapidly improving motor function,” “unable to follow two-step commands,” “prior stroke,” and “modified Rankin score (mRS)>1;” many of these are unmodifiable. Prior stroke led to exclusion of a large proportion of potentially eligible patients in our cohort. Similar findings have been reported in the European setting, 17% excluded due to “recurrent stroke” (45). Given the incidence of recurrent and silent strokes, a judicious definition of “prior stroke” is necessary to not miss out on an otherwise large majority of eligible participants. For motor function studies, “prior strokes without residual impairment” may be an acceptable definition, although these depend on the specific research questions and trial phase. Similarly, a blanket exclusion based on mRS may be impractical for stroke rehabilitation trials since nearly 20% of all moderately impaired individuals were disqualified due to this single exclusion criteria.

### In Inpatient Rehabilitation: Too Mild, Prior Stroke, Arriving to Rehabilitation Too Late for The Study

A large fraction of patients screened at inpatient rehabilitation were excluded because they were already out of the enrollment window when they arrived at the inpatient facility. CPASS enrolled patients <30-days post stroke, nearly 12% were disqualified because they could not be identified as eligible within this timeframe. Thus, it is recommended to screen eligible patients while they are still admitted to acute care; it is crucial to develop streamlined screening, chart review, and identification of potentially eligible patients at the earliest. Inability to consent due to language or aphasia led to another potentially modifiable exclusion criteria (7% in mildly impaired; 9% in severely impaired).

Nearly 62% of individuals with ischemic stroke arriving to inpatient rehabilitation were “too mild” (NIHSS motor arm item<1) for an early neurorehabilitation trial and thus disqualified from CPASS. Given the large percentage disqualified due to this single exclusion criteria, we examined patients' arm impairment using a dedicated motor function impairment test, the ARAT. ARAT evaluates fine motor function and reaching to grasp and ARAT sub-scores are highly relevant to UE stroke trials. The mean ARAT score in this cohort was 39.41±18.6 (max = 57). The minimal clinically important difference (MCID), the minimal change in score for patients to perceive an improvement in their UE motor function with the ARAT is 5.7 points (46). Thus, although classified ineligible, individuals with an NIHSS motor arm score <1 show potential for UE motor recovery and are not at the ceiling of their UE motor function. Trials may benefit from performing additional screening using a motor function-specific measure like the ARAT or SAFE for a subset of individuals. This is particularly important because prior studies have also highlighted that disqualifying “too mild” impairment in stroke trials excludes large proportions of stroke patients (41, 45). Alternately, motor-specific (pre-screening) measures such as the SAFE score have shown good predictive validity at 1 year and may be more sensitive than the NIHSS motor item score or mRS in the context of UE neurorehabilitation trials (47, 48). Similar screening data (as presented in the present report) from trials implementing the SAFE score as a prescreening measure for trial recruitment will fill an important gap for trialists. Our data on NIHSS and ARAT highlight the need for a simple, quick, motor-function specific prescreening tool that is sensitive throughout the range of UE motor impairment and shows good predictive validity for motor outcomes at 12-months or more post stroke (the typical primary outcome in post-stroke UE trials).

## Limitations

Although we present a first set of exhaustive data on trial recruitment statistics in an early subacute stroke rehabilitation trials in the US, it must be noted that these recruitment data come from a single-site phase II stroke trial. Patients were screened at a single site, an urban safety net hospital and inpatient rehabilitation facility and as such is limited to trial recruitment and patient behaviors within the US. Generalizability to other recruitment settings needs more data. Patients were screened using the motor arm item of the NIHSS, which was adequate for CPASS inclusion/exclusion criteria as shown by CPASS recruitment rates that remained comparable to similar trials, but trialists should consider adding a more sensitive motor-specific screening tool such as the SAFE score or ARAT given the relatively large numbers of patients' with demonstrated impairment on the ARAT excluded from the trial for being “too mild.”

## Conclusions

A large percentage of trial eligible patients are excluded because trialists do not get to patients in time, before discharge from acute care, and patients arrive to inpatients rehabilitation too late for trial recruitment. Trialists performing early stroke rehabilitation trials therefore need to develop streamlined screening and recruitment pipeline to engage patients while they are still in acute care. For a subset of mildly impaired patients, trialists will benefit from performing a motor function specific test like the ARAT in addition to using the NIHSS motor arm item during in-person screening for a subset of patients.

## Data Availability Statement

The raw data supporting the conclusions of this article will be made available by the authors, without undue reservation.

## Ethics Statement

The studies involving human participants were reviewed and approved by the Institutional Review Board at the MedStar Health Research Institute. The trial was registered at http://www.clinicaltrials.gov (Identifier: NCT02235974). The patients/participants provided their written informed consent to participate in this study.

## Author Contributions

SG, PF, DE, and AD conceived the research. SG conducted the research. SG, AD, and DE interpreted the findings. SG, PF, DE, and AD wrote and approved the manuscript. All authors contributed to the article and approved the submitted version.

## Conflict of Interest

The authors declare that the research was conducted in the absence of any commercial or financial relationships that could be construed as a potential conflict of interest.
